# Delivery of Surgical Services in the Coronavirus Disease Pandemic Era

**DOI:** 10.31729/jnma.5141

**Published:** 2020-06

**Authors:** Badri Man Shrestha

**Affiliations:** 1 Sheffield Kidney Institute, Sheffield Teaching Hospitals NHS Trust, Sheffield, United Kingdom.

The first case of Coronavirus Disease 2019 (COVID-19) caused by severe acute respiratory distress syndrome-Coronavirus-2 (SAR-CoV-2) was diagnosed in Wuhan, China on 31 December 2019, which has spread throughout the world and caused a global impact on the health, politics and economy. The COVID-19 pandemic continues to spread and overwhelm health systems around the world. The clinical syndrome from the COVID-19 can cause mild respiratory syndrome and fever to adult respiratory distress syndrome (ARDS) and death in severe cases.^1^ The disease has been officially classified as global pandemic by the World Health Organisation on March 11, 2020 and several countries have declared a state of emergency and imposed lockdown, social distancing, use of personal protective equipment (PPE), travel bans and other hygienic measures to mitigate the spread of disease. At the time of this writing (June 19), there were 8,559,321 confirmed COVID-19 cases worldwide with 457,190 deaths.^2^ In the epidemiological study from China with wide scale testing, 81% of the infected individuals had mild symptoms (fever, cough and malaise), 19% required hospitalisation, and 5% required critical care; with an overall mortality of 2.3%. However, case-fatality rate associated with COVID-19 was 14.9% in age > 80, 8% in the age 70-79, and 49% in critically ill patients.^3^

It is inevitable that during the COVID-19 pandemic, the surgeons and the surgical team all around the world face a unique challenge in delivering safe and optimum care to both emergency and elective surgical patients at the same time protecting both patients and health workers from potential exposure to COVID-19. Exposure to COVID-19 can occur while reviewing patients in the emergency department, some of whom may have incidental suspicious chest x-ray findings and awaiting COVID-19 swab confirmation, encountering patients with recent travel history from COVID-19 pandemic sites, and being exposed to blood or aerosol in the operating theatre that may be contaminated with the virus. The attrition of surgical workforce from illness and self-isolation can put significant strain on the available workforce that may require the staffs to fulfil alternate roles.

During this pandemic successful management has become possible through appropriate utilisation of resources and adoption of measures to mitigate transmission of COVID-19 through use of PPE, social distancing, hand hygiene, early diagnosis of COVID-19 and treatment of confirmed COVID-19 patients in designated areas. There are several detailed guidelines published from national and international organisations which provide guidance on the prioritisation of surgery during the COVID-19 crisis, which is based upon the need within specified time frame and specific measures that need to be adopted for the protection and care of patients, their surgeons and staff, and all who are served by the medical community at large.^4^

The Royal College of Surgeons of England has categorised the patients requiring surgery into five groups: (1) priority level 1a - operation needed within 24 hours; (2) priority level 1b - operation needed within 72 hours; (3) priority level 2 - surgery that can be deferred up to 4 weeks; (4) priority level 3 surgery that can be deferred up to 3 months; and (5) priority level 4 surgery that can be delated more than 3 months. The decision to prioritisation must be made cautiously as delay in instituting appropriate treatment may result in greater risk of an adverse outcome and worsening of the condition, but the team has to work within the resources available locally and nationally during the crisis.^5^ In addition, there are published guidelines pertaining to individual surgical specialties.

The Society of American Gastrointestinal and Endoscopic Surgeons (SAGES) and European Association for Endoscopic Surgeons (EAES) have made recommendations in relation to delivery of surgical services during the COVID-19 crisis. All elective surgical and endoscopic surgery should be postponed at the current time. Surgical care of the patients should be limited to those who have life-threatening conditions and patients with malignancy that could progress or with active symptoms. Others should be delayed until after peak of the pandemic is seen. This minimizes the risk to both, patient, and health care team, as well as minimizes utilization of necessary resources, such as beds, ventilators, and PPE. All non-essential staffs should work from home and minimum number of staffs should be involved face-to-face in decision-making process. All non-urgent clinic or patient visits should be postponed and should be handled remotely through telephone or video-linked consultations. All multidisciplinary meetings should be held virtually as possible and limited to team members only.^6^

With the gradual reduction of the number of COVID-19 patients in the hospital and the community, several countries are gradually resuming the surgical services in a stepwise manner. Management of the significant backlog of surgical work from increase in the number of patients on the waiting list is a major challenge. The Royal College of Surgeons of England has published its guidance on safe and efficient resumption of surgical services during and after COVID-19, which had laid emphases on acquisition of adequate hospital capacity and facilities, enhancement of workforce capacity, appropriate patient communication and reconfiguration of services.^7^

During the crisis, it is natural for the morale of the surgical team to be low, which should be restored and maintained by careful manpower planning on the frontline regardless of hierarchy, provision of an adequate work-rest cycle and giving junior member of the team time off to spend with their loved ones and family. During the COVID-19 pandemic, it is likely that some of the surgical team members may have to isolate or be on home quarantine. This period of self-isolation may provide surgeons a unique opportunity to complete research they could never find time for amidst clinical work, or simply be an opportunity for self-reflection and to delve into the deeper meaning of being the right surgeon on wrong era.^8^

In conclusion, the experience gained from the COVID-19 pandemic is evolving, we should continue to learn from the data and experiences of other countries to develop evidence-based practices. The challenge to the surgical team to deliver quality emergency and urgent surgical care continues to rise and the traditional role of surgeons remains crucial. The success relies on the development of a COVID-19 -specific infrastructure, promotion of trust among the colleagues, sharing and practical application of approved national and international guidelines to mitigate the COVID-19 pandemic.

## Conflicts of Interest:

**None.**
